# Navigating the Challenges of Persistent Left Superior Vena Cava in the Catheterization of Peripherally Inserted Central Catheter Port: A Case Study

**DOI:** 10.70352/scrj.cr.24-0088

**Published:** 2025-05-01

**Authors:** Takeshi Nakayama, Shinichiro Kobayashi, Shunsuke Murakami, Takahiro Enjoji, Hanako Tetsuo, Yusuke Inoue, Taichiro Kosaka, Akihiko Soyama, Tomohiko Adachi, Kazuma Kobayashi, Kengo Kanetaka, Susumu Eguchi

**Affiliations:** Department of Surgery, Nagasaki University Graduate School of Biomedical Sciences, Nagasaki, Nagasaki, Japan

**Keywords:** persistent left superior vena cava, peripherally inserted central catheter, 3D-CT imaging

## Abstract

**INTRODUCTION:**

Persistent left superior vena cava (PLSVC), which is asymptomatic and occurs in 0.3%–0.5% of the general population, is typically detected incidentally but can complicate cardiac procedures owing to its potential to cause arrhythmias. This condition involves an additional venous return pathway to the right atrium, which can alter the cardiac anatomy and is associated with other cardiac aortic anomalies.

**CASE PRESENTATION:**

A 75-year-old male patient required a central venous port for chemotherapy and radiation therapy for mid-thoracic esophageal cancer. Preoperative computed tomography images revealed that the PLSVC ran ventrally to the aortic and left pulmonary arteries, directly communicating with the right atrium. A peripherally inserted central catheter (PICC) port was planned. The catheter tip of the PICC port was placed within the left superior vena cava instead of the more common right superior vena cava, because the appropriate vessels could not be identified in the right upper arm. This anomaly necessitated a review of findings on the preoperative imaging and underscored the importance of early detection through echocardiography and radiographic guidance to prevent procedural complications. Reconstructed three-dimensional images and radiography-guided catheterization support the navigation of PICC port insertion.

**CONCLUSIONS:**

PLSVC, which is often asymptomatic, requires careful preprocedural planning and imaging to ensure safe PICC port insertion.

## Abbreviations

ECG
: electrocardiogram
PICC
: peripherally inserted central catheter
PLSVC
: persistent left superior vena cava
RSVC
: right superior vena cava

## INTRODUCTION

Persistent left superior vena cava (PLSVC) is a rare congenital thoracic venous anomaly that occurs in 0.3%–0.5% of the general population and 4%–12% of those with congenital heart disease.^1)^ Although frequently asymptomatic and typically detected incidentally, it can cause arrhythmia and complications during cardiac procedures. PLSVC usually drains into the right atrium, affecting approximately 20% of the left-side venous return.^2)^ Notably, PLSVC can alter cardiac anatomy, markedly enlarging the coronary sinus. It is associated with various cardiac anomalies, aortic pathologies, and heterodoxies.^3)^ Its clinical significance depends on the drainage site and the accompanying cardiac anomalies.

Central venous catheterization in patients with PLSVC poses unique challenges.^4)^ Preoperative management and intraoperative support using echocardiography and imaging are critical owing to the risks and complications associated with PLSVC, including abnormal rhythm and cyanosis.^5)^

## CASE PRESENTATION

**Fig. 1:**
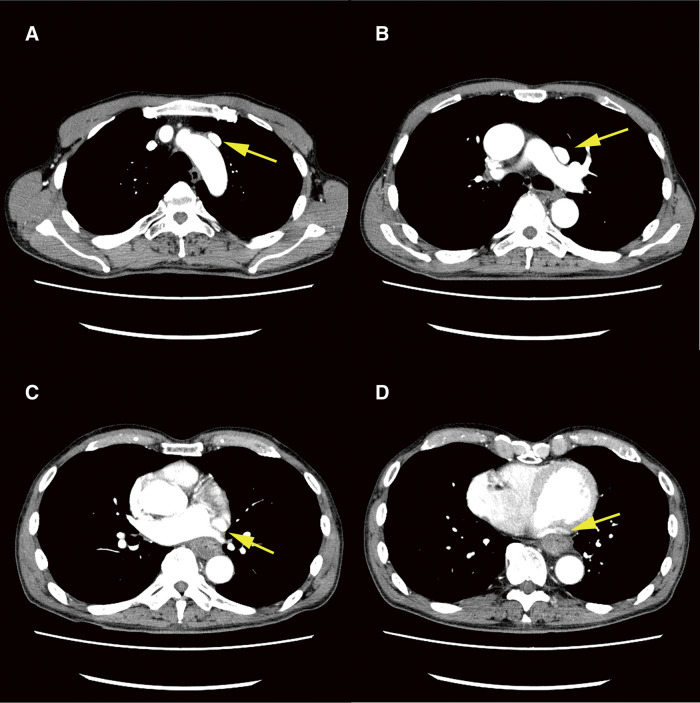
Computed tomography examination. The PLSVC runs ventral to the (**A**) aortic arch and the (**B**) left pulmonary artery. The PLSVC also communicates directly with (**C**) the right atrium from the left side of the left atrium (**D**) through the dorsal left ventricle. Yellow arrows: PLSVC. PLSVC, persistent left superior vena cava

**Fig. 2:**
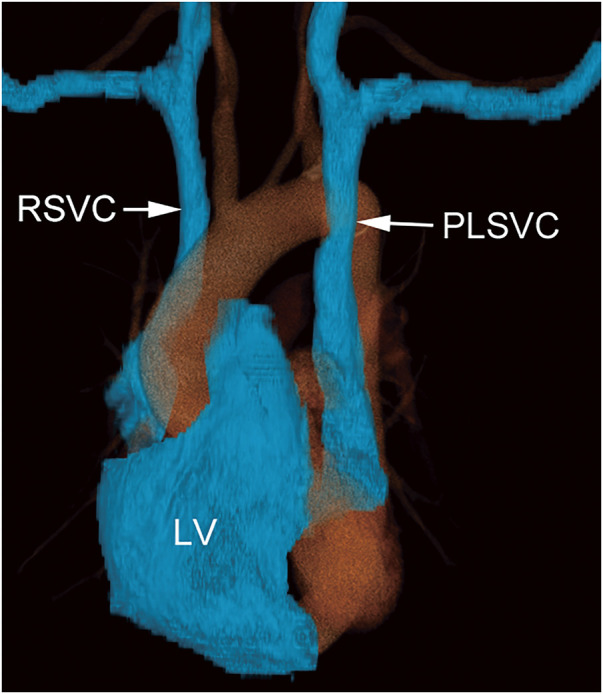
Three-dimensional reconstruction of the computed tomography image. The three-dimensional view of a CT scan from the anterior left side shows the relationship between the heart and the PLSVC. RSVC, right superior vena cava; PLSVC, persistent left superior vena cava; LV, left ventricle

**Fig. 3:**
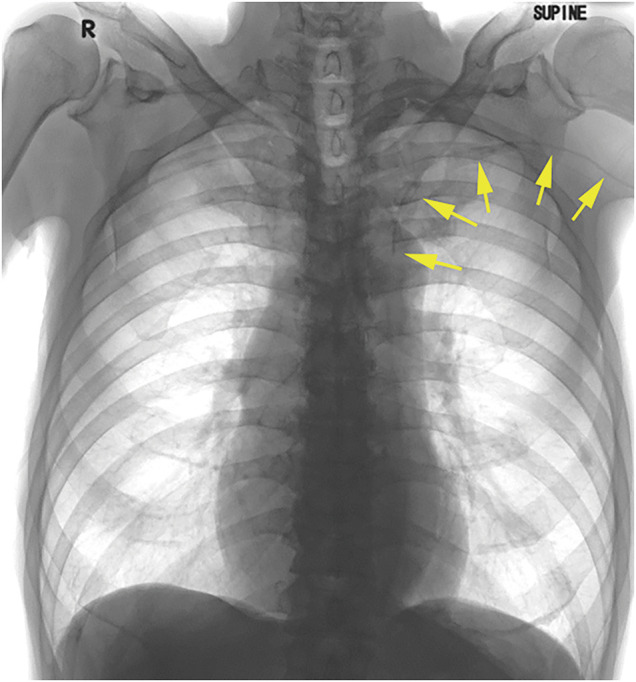
Chest radiograph after PICC port placement. The catheter tip was placed within the left superior vena cava through the PLSVC. Yellow arrows: catheter. PICC, peripherally inserted central catheter; PLSVC, persistent left superior vena cava

A 75-year-old male patient required placement of a central venous port to administer chemotherapy for mid-thoracic esophageal cancer. Radiotherapy and chemotherapy were administered. A peripherally inserted central catheter (PICC) port was used to avoid potential complications associated with cervicothoracic irradiation; a PICC port was adapted. A review of the patient’s preoperative CT images revealed that the PLSVC ran ventral to the aortic arch, and left pulmonary artery, communicating directly with the right atrium (**Fig. 1**). A 3D-reconstructed CT image view from the anterior left side revealed the relationship between the heart and the PLSVC (**Fig. 2**). The left side was selected for PICC port insertion based on the patient’s preference, as the patient was right-handed, and the appropriate vessels could not be identified in the right upper arm. A catheter was inserted into the left upper arm through the cephalic vein. During the procedure, the guidewire, observed under radiography-guided catheterization, took an atypical path through the thorax and the left mediastinum. The catheter tip was placed within the left superior vena cava instead of the commonly used right superior vena cava (RSVC) (**Fig. 3**). The PICC port remained stable for 3 months post-procedure without any complications.

## DISCUSSION

PLSVC is a congenital anomaly in which the left anterior cardinal vein does not regress, resulting in the formation of an additional large vein. It is the most common abnormality of the major thoracic veins, with a prevalence of 0.3%–0.5% in the general population.^3)^ PLSVC varies in vascularity and can be classified into four types.^6)^ Approximately 0.07%–0.13% of PLSVCs lack the RSVC, while 80%–90% have equally developed RSVCs and PLSVCs.^7)^ In the current case, the vascular structure was identified as Type IIIb, characterized by equally developed RSVC and PLSVC. Typically, asymptomatic PLSVCs are discovered incidentally during invasive procedures such as central venous catheter or pacemaker implantation.^8,9)^ Diagnostic imaging is especially valuable for ensuring safe catheter placement in patients with vascular anomalies such as PLSVC. Pre-existing imaging studies frequently provide essential information for planning catheter placement in patients requiring long-term central venous access to administer chemotherapy or nutritional support. However, routine imaging of all patients undergoing short-term central venous catheterization may not be necessary, given the low incidence of such anomalies and concerns regarding radiation exposure.

PICC placement can be complicated by unexpected deviation of the catheter into unusual venous pathways, such as the PLSVC or minor veins near the innominate vein.^3,10)^ In the current case, the catheters used in the central venous system deviated from the PLSVC. In patients with PLSVC, safe catheter placement often requires careful imaging and verification because normal anatomical landmarks may be misleading.^11)^ Cannulation site preference shifts toward the left internal jugular vein in cases of PLSVC, particularly in the absence of the RSVC.^4)^ If a PLSVC is present, the catheter path may differ from the usual route, necessitating additional imaging for confirmation. Thus, the reconstructed PLSVC image before the PICC port placement is useful. A 3D-reconstructed CT image offers superior spatial resolution, making it an invaluable tool for the rapid and precise diagnosis of anatomical anomalies.^12)^ Unlike conventional radiography or plain CT, which may struggle to delineate aberrant vascular pathways, 3D CT enables detailed 3D visualization of vascular structures.^13,14)^ This capability is crucial for reducing the risks associated with catheter placement and preventing procedure-related complications. Moreover, in certain PLSVC cases, venous drainage bypasses the coronary sinus and flows directly into the left atrium, substantially increasing the risk of venous thrombosis and embolism.^15)^ The application of 3D-CT facilitates comprehensive preoperative assessment of such hemodynamic abnormalities, providing critical information essential for formulating an optimal strategy for PICC port placement.

It is well-recognized that the ratio of catheter diameter to vein diameter markedly influences the risk of thrombosis during central venous catheter placement. Maintaining a ratio below 45% is considered crucial for ensuring safe blood flow and minimizing complications.^16)^ In the current case, we used a PICC port, which generally has a smaller diameter than conventional central venous catheters. Specifically, the PICC port catheter used in our case had an outer diameter of 1.7 mm, while the PLSVC measured 13.3 mm in diameter, resulting in a catheter-to-vein ratio of approximately 12.8%, sufficiently lower than the recommended threshold of 45%. Given that the risk of thrombosis is closely associated with the catheter size, the use of a PICC port in patients with PLSVC may be advantageous for reducing thrombotic complications.

Radiography-guided catheterization is recommended in cases with anatomical variations to identify and confirm the vascular structures before central venous port placement.^17)^ It is important to confirm the end position of the catheter using radiography in patients with PLSVC, especially to avoid complications associated with misplacement, such as thrombosis or arrhythmias.^18)^ Therefore, PICC port placement via the right cephalic vein should be considered in cases of PLSVC. However, our patient was right-handed, and no suitable vessels could be identified in the right upper arm. The intracavitary electrocardiogram (ECG) also contributes to the existing literature by providing clinical evidence of the feasibility and safety of venous port insertion in patients with PLSVC.^19)^ The specific ECG pattern during catheter insertion in patients with PLSVC is a small negative P-wave in lead II.^19)^ In the current case, no change in ECG was observed because the catheter tip was placed within the left superior vena cava.

## CONCLUSIONS

PLSVC is frequently asymptomatic and can be detected during PICC port insertion. Its presence poses unique challenges for safe catheter placement, necessitating careful preprocedural planning and verification of catheter position. Radiography-guided catheterization and awareness of vascular anomalies on 3D-CT imaging are key to ensuring patient safety and successful placement of the PICC port.

## ACKNOWLEDGMENTS

The authors extend their gratitude to ChatGPT-4o and Consensus-beta for their role in editing this manuscript and to Paperpal for assisting with the refinement of the English language presentation. They also thank Editage (www.editage.jp) for the English language editing.

## DECLARATIONS

### Funding

This research was supported by a JSPS Grant-in-Aid for Early-Career Scientists (Grant No. 21K16400), underscoring our commitment to advancing medical knowledge and practice.

### Authors’ contributions

All the authors have read and approved the final manuscript.

All authors agree to take responsibility for all aspects of this study.

TN and SK collected the patient data and edited the manuscript.

SK approved the final submission of this manuscript.

SM participated in surgical planning and surgery.

KKa and SE supervised patient treatment.

HT and TE participated in discussions.

YI, TK, AS, TA, and KKo developed patient care plans.

### Availability of data and materials

The datasets analyzed during the current study are not publicly available but can be provided upon reasonable request.

### Ethics approval and consent to participate

This investigation was conducted in strict adherence to the ethical guidelines of our institution, ensuring the utmost respect for the patient’s rights and privacy. This study was approved by our commitment to ethical research practices and patient confidentiality (Approval No.: 24052010).

### Consent for publication

Informed consent for the publication of this case study was obtained from the patient.

### Competing interest

The authors declare no conflicts of interest and ensure the impartiality and integrity of this study.
